# The vampyrellid amoeba *Strigomyxa ruptor* gen. et sp. nov. and its remarkable strategy to acquire algal cell contents

**DOI:** 10.1002/ece3.70191

**Published:** 2024-08-28

**Authors:** Andreas Suthaus, Sebastian Hess

**Affiliations:** ^1^ Institute for Zoology University of Cologne Cologne Germany; ^2^ Division for Biology of Algae and Protozoa, Department of Biology Technical University of Darmstadt Darmstadt Germany

**Keywords:** algae, amoebae, Cercozoa, Endomyxa, Rhizaria, Zygnematophyceae

## Abstract

The vampire amoebae (Vampyrellida, Rhizaria) inhabit freshwater, marine, and terrestrial ecosystems and consume a wide range of eukaryotic prey. This includes diverse microalgae, fungi, and microscopic animals. One of the most captivating aspects of the vampyrellids is their ability to extract the cell contents of other eukaryotes after local dissolution of the prey cell wall, a feeding strategy that occurs in several vampyrellid families, but is best studied in *Vampyrella* species that attack zygnematophyte green algae. Here, we report two new vampyrellid strains from temperate moorlands in Germany with a yet‐undescribed feeding strategy: internal protoplast extraction and cell wall regurgitation. This feeding strategy involves the phagocytosis of whole desmid cells (genus *Closterium*, Zygnematophyceae), internal cleavage of the algal cell wall, extraction of the cell contents, and subsequent exocytosis of bundled empty cell walls. The large primary food vacuole formed during the process has exceptional functions, as it forms internal feeding pseudopodia, packages algal cell contents into smaller secondary vacuoles, and transforms into a “waste vacuole” with cell wall remnants. The new feeding strategy, which – in the widest sense – is reminiscent of the pellet casting of owls, reveals a stunningly sophisticated behavior of single protist cells. Based on morphological, phylogenetic, and autecological data, both vampyrellid strains are nearly identical and here assigned to a new and quite unique vampyrellid taxon, *Strigomyxa ruptor* gen. et sp. nov. (Leptophryidae, Vampyrellida).

## Graphical abstract

The new vampyrellid amoeba *Strigomyxa ruptor* phagocytizes algal cells and opens them internally to extract the cell contents. Afterward, the algal cell walls are discarded through a “waste vacuole.” This sophisticated feeding strategy, here termed “internal protoplast extraction and cell wall regurgitation,” exemplifies the extraordinary complexity of prey manipulation found in protists.

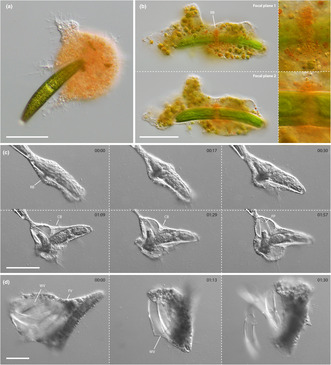

## 1 INTRODUCTION

**FIGURE 1:**
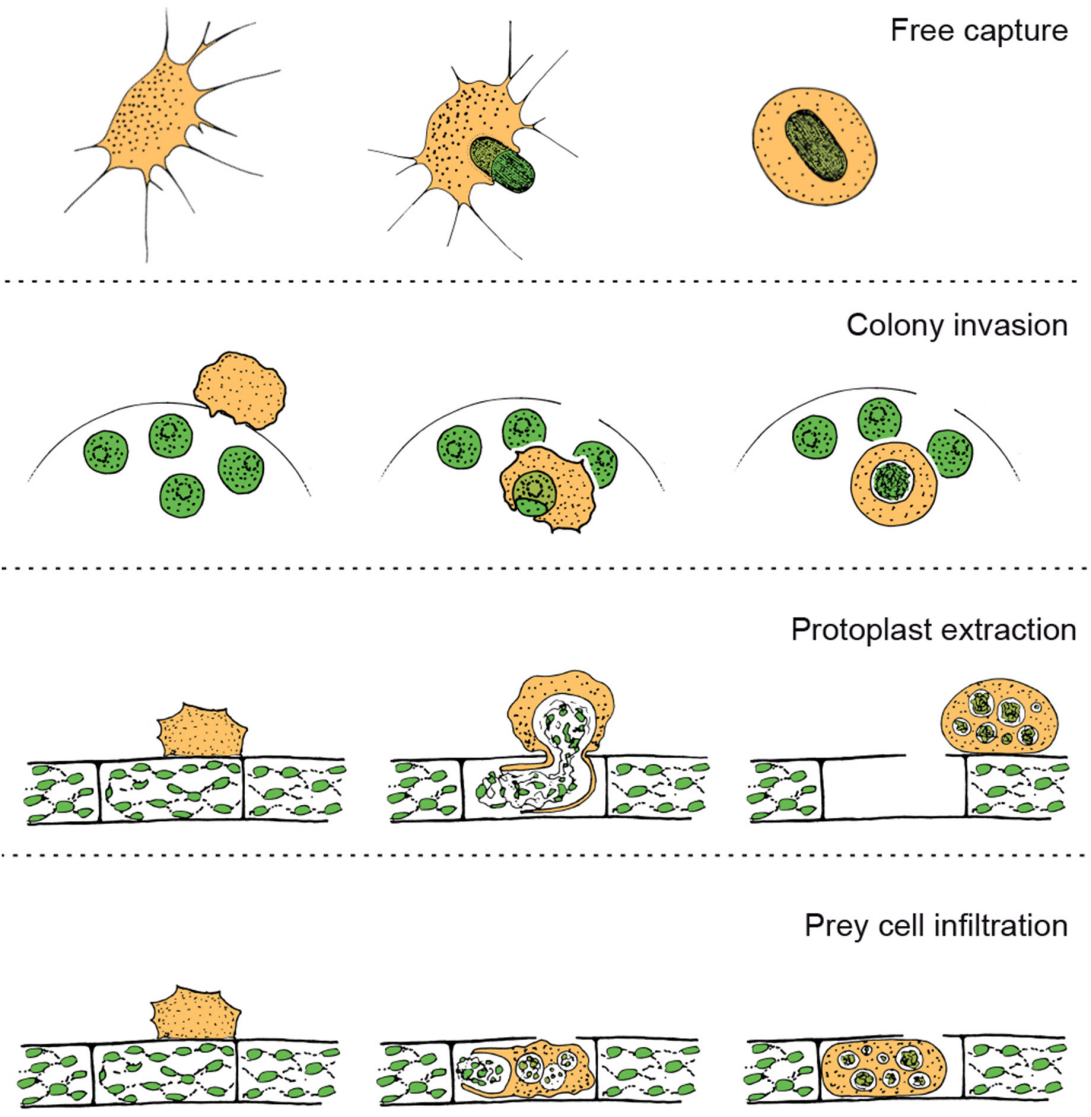
Known feeding strategies of vampyrellid amoebae (from Hess & Suthaus, 2022, modified).

The order Vampyrellida comprises a lineage of ecologically diverse, predatory amoebae within the Rhizaria supergroup of eukaryotes (Bass et al., 2009; Hess et al., 2012). All known representatives are naked (without any cell coverings), exhibit a tendency to form filose pseudopodia, and possess a two‐part life history, comprised of the amoeboid stage (=trophozoite) and an obligatory digestive cyst stage (Hess & Suthaus, 2022). As revealed by SSU rRNA gene phylogenies, the vampyrellids can be subdivided in several family‐level clades, of which only five contain species with phenotypic information. The other three clades are exclusively known from environmental sequencing studies (Berney et al., 2013). Vampyrellids are globally distributed as they have been found on six continents as well as in the oceans (Berney et al., 2013; Gong et al., 2015; Hess et al., 2012; Homma & Kegasawa, 1984; Lentendu et al., 2018; Old & Oros, 1980; Vimercati et al., 2019). They inhabit freshwater, marine, and soil ecosystems and interact with a variety of other organisms. Almost all of the ~50 known vampyrellid species feed on other eukaryotes, including, for example, green algae, cryptomonads, diatoms, heterotrophic flagellates, ciliates, fungi (including spores), and microscopic animals, such as nematodes and rotifers (Bass et al., 2009; Cienkowski, 1865; Dobell, 1913; Grell, 1985; Hess, 2017b; Hess et al., 2012; Valkanov, 1970; Weber et al., 1952; Zopf, 1885). While some vampyrellids appear to have a very narrow prey range and are considered specialists (e.g., *Vampyrella* species), others are rather generalist predators (e.g., *Leptophrys vorax*; Cienkowski, 1865; Hess, 2017a, 2017b; Hess et al., 2012; Hülsmann, 1993; Zopf, 1885). To acquire and consume eukaryotic prey of diverse structure and biochemistry, vampyrellids evolved several feeding strategies (Figure 1; Hess & Suthaus, 2022). Most notable is the extraction of foreign cell contents, which certainly inspired the name of the first described genus, *Vampyrella* (Cienkowski, 1865). This feeding strategy, referred to as “protoplast extraction,” allows vampyrellids to overcome the natural barriers of bulky or indigestible prey, for example, the cellulosic cell walls of green algae (Hess, 2017a; Hess et al., 2012; Suthaus & Hess, 2023). However, there are three other feeding strategies known, namely, “free capture,” “colony invasion,” and “prey infiltration” (Figure 1). These feeding strategies are not mutually exclusive, as some generalist vampyrellids (e.g., *Sericomyxa perlucida*) can switch their strategy depending on the prey type (More et al., 2021). Overall, vampyrellid amoebae display a stunning and exceptional diversity of predator–prey interactions, by far exceeding those found in many other orders of phagotrophic protists. And yet, we only scratched the surface, as most of the known vampyrellid genotypes are uncharacterized and we keep discovering new vampyrellid species.

We discovered two new vampyrellid strains from German moorlands, which consume desmidiacean green algae of the genus *Closterium* by a hitherto unknown process. In this study, the amoebae were characterized by life history observations, various microscopy techniques, feeding experiments, and molecular phylogenetics. Based on unique cellular and behavioral features, and a distinct position in the vampyrellid SSU rRNA gene phylogeny, we introduce *Strigomyxa ruptor* gen. et sp. nov. with a novel vampyrellid feeding strategy.

## 2 MATERIALS AND METHODS

### 2.1 Establishment and maintenance of cultures

Samples of sediment were taken from two moorland waters, namely, the main pond of the moor of Thielenbruch, Cologne, Germany (50.987959, 7.078928; autumn of 2021), and the Heideweiher of the nature reserve “Heiliges Meer,” Recke, Germany (52.345986, 7.621127; autumn of 2021). Samples with vampyrellid cysts were enriched with cultivated *Closterium* species (strains C120_SH, C250_SH, CCAC1125) as potential food and monitored with a Motic AE2000 inverted microscope (Motic, Hong Kong) for several days. Motile vampyrellid cells were isolated with a glass micropipette, transferred to MilliQ water with *Closterium* sp. (strain C120_SH) and let grow for several days. Growing vampyrellid populations were transferred to 150‐mL Erlenmeyer flasks or T25 culture flasks with rectangular neck and vented cap (Flacon, Corning, NY, USA) and maintained by regular feeding with *Closterium* sp. (strain C120_SH, interval of about 3 weeks). The resulting vampyrellid strains, SR.01 from Thielenbruch and SR.02 from the nature reserve Heiliges Meer, were kept at 15°C without light. They are available from the corresponding author (S.H.) upon request. Algal strains were grown at the same temperature in Waris‐H medium, a mineral salt medium with vitamins and HEPES buffer (McFadden & Melkonian, 1986), under a 14/10 h light/dark cycle with 10–30 μmol photons m^−2^ s^−1^. The algal cultures are available from the corresponding author (strains without CCAC numbers) or the Central Collection of Algal Cultures (https://www.uni‐due.de/biology/ccac/).

### 2.2 Light and fluorescence microscopy

Basic light microscopy and time‐lapse photography were done with the Motic AE2000 inverted microscope (Motic Hong Kong Limited, Hong Kong) equipped with brightfield and phase contrast optics and with a MikroLive 6.4MP CMOS camera (MikroLive, Oppenau). Differential interference contrast (DIC) microscopy and fluorescence microscopy were done with the ZEISS Axio Observer 7 inverted microscope (ZEISS, Oberkochen, Germany), equipped with differential interference contrast optics, digital camera (ZEISS Axiocam 512 color), as well as the Plan‐Neofluar 20×/0.5, Plan‐Neofluar 40×/1.3, and Plan‐Neofluar 100×/1.3 objectives. For observation of motility and feeding at high magnification, an orange interference filter (transmission 589 nm ± 10 nm) was used to prevent unwanted reactions (e.g., hypnocyst formation) and damage of the cells. Cell dimensions were measured with Fiji (Schindelin et al., 2012). Nuclei of live cells were stained with Hoechst 33528 (Invitrogen, Waltham, Massachusetts, USA) at 1 μg mL^−1^ for five minutes at room temperature and then imaged with the ZEISS Colibri 5 LED illumination system (RGB‐UV) with the following filter set: 96 HE BFP (excitation 390/40, emission 450/40). Emptied *Closterium* cells were stained with 0.02% Calcofluor White (Sigma Aldrich, Missouri, USA) in water for 10 min, and then imaged with the same fluorescence microscopy setup. Adobe Photoshop CS4 (Adobe Systems, Munich, Germany) was used to adjust color balance and contrast of light micrographs.

### 2.3 Confocal laser scanning microscopy and λ‐scans

A Leica TCS SPE confocal laser scanning microscope (Leica Microsystems, Wetzlar, Germany) was used to acquire λ‐scans in the “xyλ mode” with a 405 nm laser for excitation. After defining a region of interest (ROI: part of an empty digestive cyst) and a reference area (background), measurements were done from 410 to 610 nm with a detection bandwidth and step size of 10 nm. To correct for background fluorescence, the reference values (background) were subtracted from the emission values (ROI). The corrected signal values were used for display.

### 2.4 Feeding experiments

Algal cultures of 29 zygnematophytes and *Oedogonium stellatum* (Chlorophyta) were grown in Waris‐H medium at 15°C with a 14/10 h light/dark cycle at 10–30 μmol photons m^−2^ s^−1^. Once grown to suitable density, 1 mL of algal culture was suspended in sterile water in small Petri dishes and inoculated with starving trophozoites of the vampyrellids. Both vampyrellid strains (SR.01 and SR.02) were tested, to ascertain potential differences in prey spectrum. The experimental cultures were kept at room temperature in dim light and monitored daily for three weeks to determine whether the vampyrellids fed were growing and growth was persistent. Feeding was identified based on the presence of emptied prey cells and the presence of digestive cysts. The results were summarized in two categories, namely, “feeding and growth” (+) and “no feeding and no growth” (−).

### 2.5 DNA amplification, sequencing, and sequence assembly

Starving trophozoites were isolated from cultures with a glass micropipette, passed through several washing steps in nuclease‐free water, transferred into 4 μL REPLI‐g Advanced sc Storage Buffer (Qiagen GmbH, Hilden, Germany), and then frozen at −20°C prior to whole genome amplification with the REPLI‐g Advanced Single Cell Kit (Qiagen GmbH, Hilden, Germany). After amplification, the resulting DNA was diluted 1:100 and used as a template for polymerase chain reaction (PCR) to amplify the nuclear SSU rRNA gene with the DreamTaq™ DNA Polymerase (Fermentas, St. Leon‐Rot, Germany). We used the universal eukaryotic primers EukA and EukB (Medlin et al., 1988) as previously described (Suthaus & Hess, 2023). Positive PCR products were purified using the NucleoSpin Gel and PCR Clean‐up kit (Macherey‐Nagel, Düren, Germany) and subjected to commercial Sanger sequencing by Eurofins Genomics (Eurofins Scientific, Luxembourg). The resulting sequences were assembled using the AlignIR software (LI‐COR Biosciences, Nebraska, United States). As the sequences of strains SR.01 and SR.02 were identical, we deposited a single SSU rRNA gene sequence of *S. ruptor* at GenBank (accession number PP769605).

### 2.6 Alignments and phylogenetic analyses

BLAST searches of the SSU rRNA gene sequence of *S. ruptor* with the nr/nt database (https://blast.ncbi.nlm.nih.gov/Blast.cgi) returned hits with high genetic identity from vampyrellid amoebae (e.g., *Theratromyxa weberi* GQ377666, 95.01%). The new sequence was added to a previously published alignment of vampyrellid amoebae (Suthaus & Hess, 2023) and aligned with MUSCLE in Seaview 5.0.4 (Gouy et al., 2021), followed by manual curation. Clearly aligned sites were manually selected, and the resulting dataset (92 sequences, 1498 sites) was subjected to phylogenetic analyses. We performed maximum likelihood (ML) analyses with raxmlGUI 2.0.5 using the model GTR + Γ + I and 100 runs plus 1000 thorough bootstrap replicates (Edler et al., 2021). Additionally, we performed Bayesian Inference (BI) with Beast 2.7.3. (Bouckaert et al., 2014) using the model GTR + Γ + I, 5,000,000 generations (trees sampled every 1000 generations) and a 25% burn‐in (1,250,000 generations discarded). Stationarity of the BI was confirmed using Tracer 1.7.2. (Rambaut et al., 2018).

### 2.7 Generation of type material

Cultures of *S. ruptor* (strain SR.01) were grown in small Petri dishes (55 mm). Once suitably dense, the cells were fixed in the Petri dish with 2.5% glutaraldehyde in MT buffer (30 mM HEPES, 15 mM KCl, 5 mM MgSO_4_, 5 mM EGTA, 100 μM DTT, pH 7.0 adjusted with KOH) for 20 min at room temperature. Glutaraldehyde was removed by washing with water, followed by post‐fixation with 0.04% osmium tetroxide in water for 20 min. The cells were subsequently washed twice with water, scraped off the Petri dish with a cell scraper, and let settle on poly‐L‐lysine‐coated coverslips for 30 min. Afterward, the coverslips were centrifuged for 10 min at 1000 *g* with slow acceleration and deceleration and passed through a series of ethanol/water mixtures (20–40–60–80–90–100% ethanol, 5 min each). The ethanol was then replaced by 100% isopropanol, followed by an incubation for 20 min. After removal of excess fluid from the cover slips, the latter were mounted with a droplet of a 1:1 Euparal/isopropanol mixture. The Euparal resin (Carl Roth, Karlsruhe, Germany) was allowed to harden for at least 24 h before examination.

### 2.8 Nomenclatural acts

This published work and the nomenclatural acts it contains have been registered in ZooBank (http://zoobank.org/), the proposed online registration system for the ICZN. The ZooBank LSIDs (Life Science Identifiers) can be resolved, and the associated information can be viewed through any standard web browser by appending the LSID to the prefix “http://zoobank.org/.” The LSID for this publication is as follows: urn:lsid:zoobank.org:pub:AA297467‐5F92‐4DE3‐ACA3‐B42BCB71B3D9.

## 3 RESULTS

### 3.1 Natural observations

In natural samples from ponds of two moorlands in Germany (see Methods for geographic details), we observed relatively large, roundish digestive cysts of an unidentified vampyrellid. These cysts had no visible prey cells inside and a strong orange‐red color, pointing to a species that feeds on the protoplasm of green algae. However, in the samples we did not find any algal cells with distinct cell wall perforations as known from described vampyrellids (Suthaus & Hess, 2023). Instead, we noticed that the indigenous population of undetermined *Closterium* species (Desmidiales, Zygnematophyceae) decreased, while empty semicells of these desmids accumulated. Time‐lapse microscopy on the natural samples finally revealed that the vampyrellid amoebae associated with the observed cysts engulf entire *Closterium* cells and eject empty semicells during an unexpected feeding process (Video 1). With this information, we were able to establish two laboratory strains (SR.01, SR.02) of a new vampyrellid taxon, referred to as *Strigomyxa ruptor* in the following. The detailed morphological analyses have been done on strain SR.01 only, as we did not recognize any significant differences between the two strains in our cultures (note a 100% sequence identity of the SSU rRNA genes, details below).

**VIDEO 1:** Time‐lapse observation of the unknown vampyrellid in a natural sample from the Thielenbruch moorland; brightfield, 600× natural speed.

### 3.2 Morphology and motility of the amoebae

**FIGURE 2:**
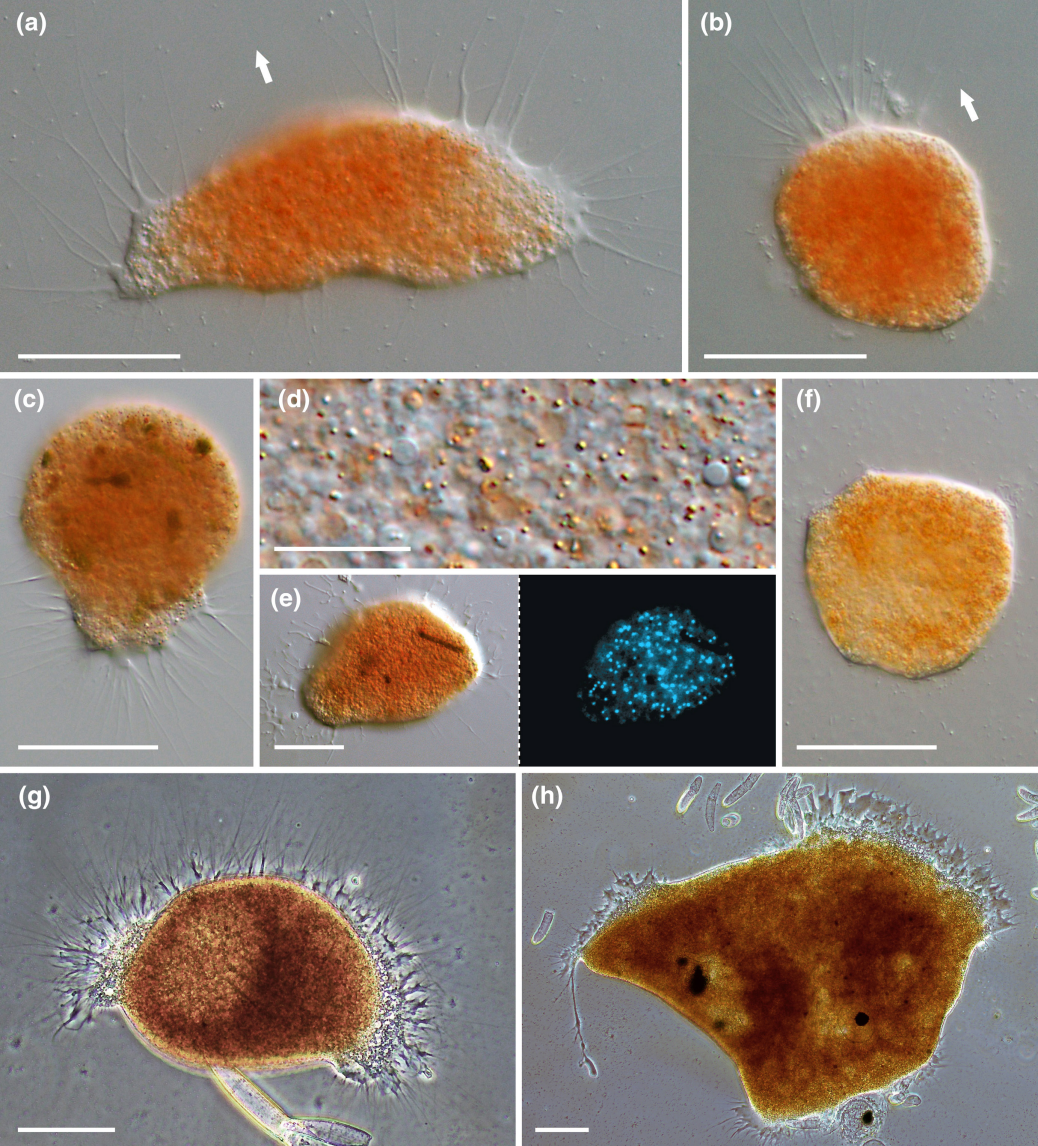
Morphological characteristics of *Strigomyxa ruptor*; DIC (a–f), fluorescence (f) and phase contrast (g, h). (a) Expanded trophozoite. White arrow indicates direction of movement. (b) Trophozoite with spherical cell body and anterior filopodia. White arrow indicates direction of movement. (c) Trophozoite with filopodia attached to substrate and spherical cell body. (d) Cytoplasm of squashed cell. (e) Hoechst‐stained trophozoite with multiple nuclei (left = DIC, right = fluorescence). (f) Hypnocyst after light exposure. (g, h) Plasmodia crawling along substrate in late‐stage culture. Scale bars: 50 μm (a–c, e, f), 10 μm (d), 100 μm (g, h).

The amoebae of *S. ruptor* ranged between 50 and 150 μm in size, with dynamic and variable cell bodies (Figure 2a). They were usually attached to the substrate and thus represented most closely the expanded morphotype as defined by Hess et al. (2012). However, the vampyrellid occasionally presented with a more or less spherical cell body and filopodia at the anterior cell portion, extended in the direction of movement (Figure 2b). In some instances, these filopodia originated from a pseudopodial “foot,” where the filopodia remained in contact with the substrate while the spherical cell body extended upward (Figure 2c). The main cell body possessed red, granular cytoplasm and, in some instances, a distinct, hyaline ectoplasm from which the filopodia emerged. The filopodia were without any granules, tapering, and frequently forked at the base (Figure 2a–c). They measured up to about 70 μm when fully extended. The cytoplasm of *S. ruptor* was very opaque due to numerous cellular inclusions (Figure 2d). Even though it was not possible to unambiguously identify nuclei by conventional DIC microscopy, we were able to visualize several dozens of minute nuclei by fluorescence microscopy of Hoechst‐stained cells (Figure 2e). These nuclei were scattered throughout the cytoplasm and measured up to 2 μm. The cells of *S. ruptor* turned out to be exceptionally photosensitive as compared with other vampyrellid species (e.g., *Pseudovampyrella closterii*, *Vampyrella lateritia*). Short periods (30 s) of white light in standard DIC microscopy induced the retraction of filopodia, the formation of a hypnocyst (Figure 2f) and, in some cases, even cell rupture. We used this information for our live observations on the feeding process and applied orange monochromatic light in some applications (see below). As typical for several other vampyrellid species, *S. ruptor* could form plasmodia through the fusion of trophozoites and split again into smaller units. The plasmodia most frequently exhibited a compact morphology but could also be fan‐ or ribbon‐shaped (Figure 2g,h). Plasmodia formation was prevalent in older cultures with depleted food, sometimes resulting in cells that exceeded 1 mm in size.

### 3.3 Prey range specificity and feeding process

**FIGURE 3:**
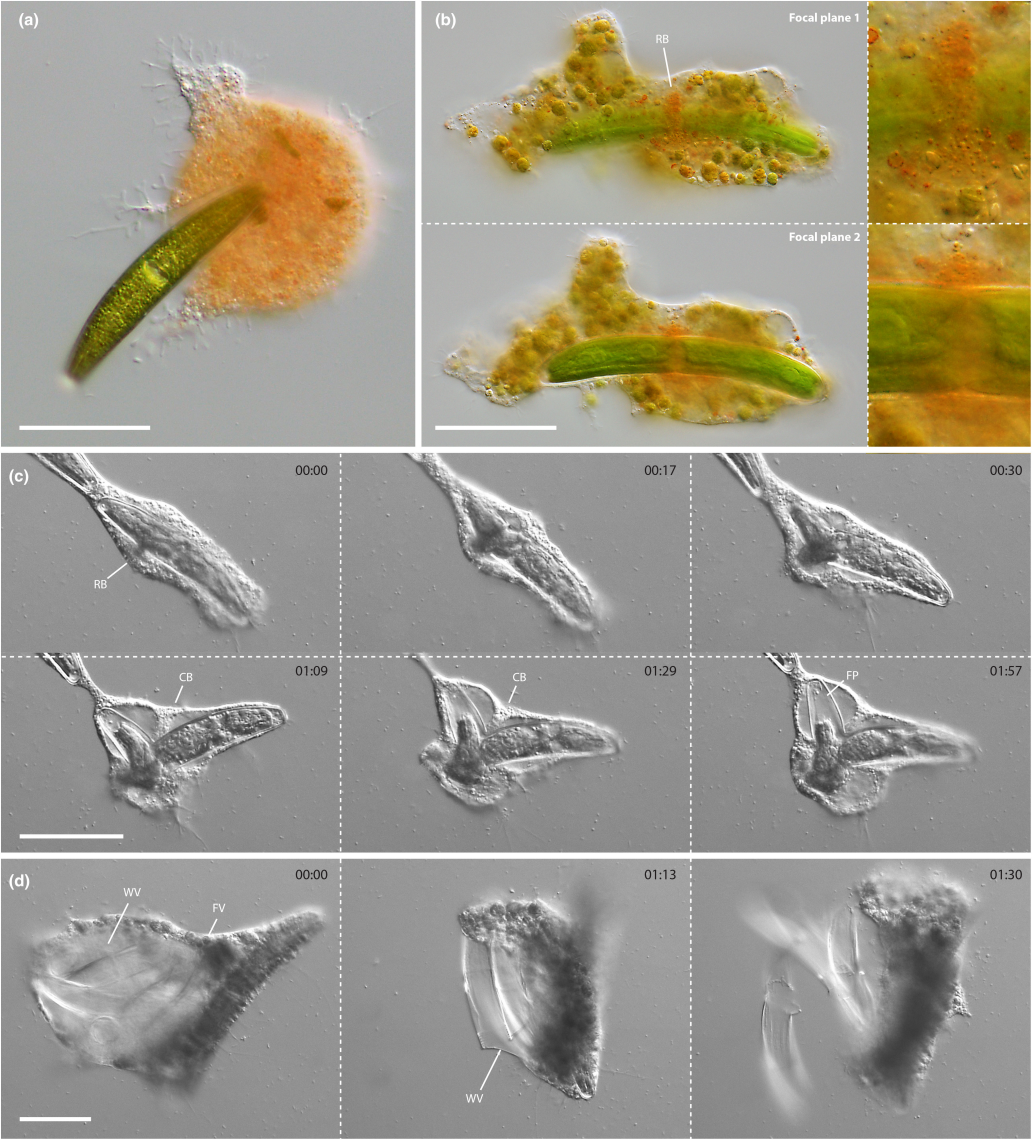
Feeding process of *Strigomyxa ruptor*; DIC (a–d), with orange filter and monochromatic image capture (c, d). (a) Trophozoite engulfing a *Closterium* cell. (b) Reddish band of granular cytoplasm (RB) prior to cell wall cleavage in two focal planes. Right panels show the same structure at higher magnification. (c) Time series of cell wall cleavage and protoplast extraction. Note the reddish band (RB), cytoplasmic brace (CB), and the feeding pseudopodium (FP). Time stamps are shown in mm:ss. (d) Time series showing the burst of the large waste vacuole (WV) and release of remnant algal cell walls. Note that the cell contents of the algae remain in small (secondary) food vacuoles (FV). Scale bars: 50 μm.

The feeding experiments with a range of zygnematophyte green algae and the chlorophyte *Oedogonium stellatum* revealed that *S. ruptor* consumed exclusively *Closterium* species (Table 1). Seven out of the 22 tested *Closterium* strains were suitable prey, namely, strains that were identified as or similar to *C. intermedium*, *C. striolatum*, and *C. closterioides*. Independent of the prey species, *S. ruptor* used the same feeding strategy. The trophozoites incorporated entire *Closterium* cells by phagocytosis (Figure 3a) and often collected several cells by frequent expansion of the cell body (Video 2). The number of prey cells in a trophozoite ranged from one to seven and appeared to be dependent on the size ratio between predator and prey (Note that plasmodia feed as well and might be able to take up more prey cells). Each prey cell was taken up in a separate food vacuole and the vacuolar membrane surrounded the prey very closely as it was not discernible during this phase. After phagocytosis, a red band of granular cytoplasm formed around the *Closterium* cell, frequently, but not always, in the central region of the algal cell (Figure 3b). This band, which did not extend around the entire circumference of the alga, appeared to be involved in the local breakdown of the algal cell wall. After a short timeframe, the *Closterium* cell ruptured exactly at the location of the red band and some algal protoplasm erupted in an initial burst, probably driven by the turgor pressure of the alga (Figure 3c, Video 3). Once this had subsided, the algal protoplasm was packaged into smaller food vacuoles and the amoeba extracted the remaining cell contents of the alga with a hyaline feeding pseudopodium (Figure 3c). During this process, the food vacuole expanded around the cleaved *Closterium* cell and the two halves of the ruptured alga were bent into a V‐like shape. However, the vampyrellid still contacted the outside of the algal cell immediately opposite the cleavage site by a cytoplasmic protuberance into the vacuole (Figure 3c). While the “secondary” food vacuoles with algal cell contents were distributed throughout the vampyrellid cell body, the emptied algal walls were merged into a large “waste vacuole” (Figure 3d). After an undetermined period of time, the waste vacuole fused with the plasma membrane and the trophozoite discarded the remnant *Closterium* cell walls (Figure 3d). As *S. ruptor* collected several prey cells during the active feeding phase, we observed several instances of end‐stage feeding on multiple engulfed algae. The duration of the feeding process was strongly dependent on the amount of prey consumed as well as on the prey size. However, engulfment, cleavage, extraction, and ejection of cell walls for a single *Closterium* sp. cell (strain C120_SH) took about 30–45 min.

**TABLE 1:** Results of the feeding experiment with *Strigomyxa ruptor* categorized in “feeding and growth” (+) and “no feeding and no growth” (−).

Algal species	Algal strain	SR.01	SR.02
*Closterium abruptum*	C007	−	−
*Closterium baillyanum crassum*	D249	−	−
*Closterium* cf. *intermedium* (250)	C250_SH	+	+
*Closterium* cf. *pseudolunula*	D459	−	−
*Closterium* cf. *striolatum*	C009	+	+
*Closterium closterioides* var. *closterioides*	Sch24	+	+
*Closterium closterioides* var. *closterioides*	D084	−	−
*Closterium cornu*	CCAC 1125	−	−
*Closterium costatum*	C084	−	−
*Closterium cynthia* var. *Cynthia*	D022	−	−
*Closterium didymotocum*	C013	−	−
*Closterium gracile*	Cgrac01_SH	−	−
*Closterium intermedium*	C035a	+	+
*Closterium intermedium*	C450	+	+
*Closterium limneticum*	CCAC 2687	−	−
*Closterium littorale*	C290	−	−
*Closterium moniliferum*	F168	−	−
*Closterium pritchardianum*	D050	−	−
*Closterium* sp. (120–160)	C120_SH	+	+
*Closterium striolatum*	C459	+	+
*Euastrum humerosum*	N022	−	−
*Euastrum oblongum*	N058	−	−
*Micrasterias americana*	C514	−	−
*Micrasterias rotata*	D465	−	−
*Netrium digitus*	Ndigi_01_AS	−	−
*Oedogonium stellatum*	CCAC 2231 B	−	−
*Penium margaritaceum*	CCAC 0215	−	−
*Pleurotaenium trabecula*	N059	−	−
*Zygnema pseudogeanum*	CCAC 0199	−	−

*Note*: Most microalgae were identified by Dr. Karl‐Heinz Linne von Berg and by the authors with an identification key (Lenzenweger, 1996), sometimes tentatively. Strains that could not be identified to the species level based on morphology were kept unidentified.

**VIDEO 2:** Feeding process of *Strigomyxa ruptor*, strain SR.01; brightfield, 60× natural speed. The amoeba takes up three cells of *Closterium intermedium*, extracts the cell contents, and discards the emptied cell walls.

**VIDEO 3:** Close‐up examination of cell wall cleavage and protoplast extraction in *Strigomyxa ruptor*, strain SR.01; DIC; 12× natural speed.

**FIGURE 4:**
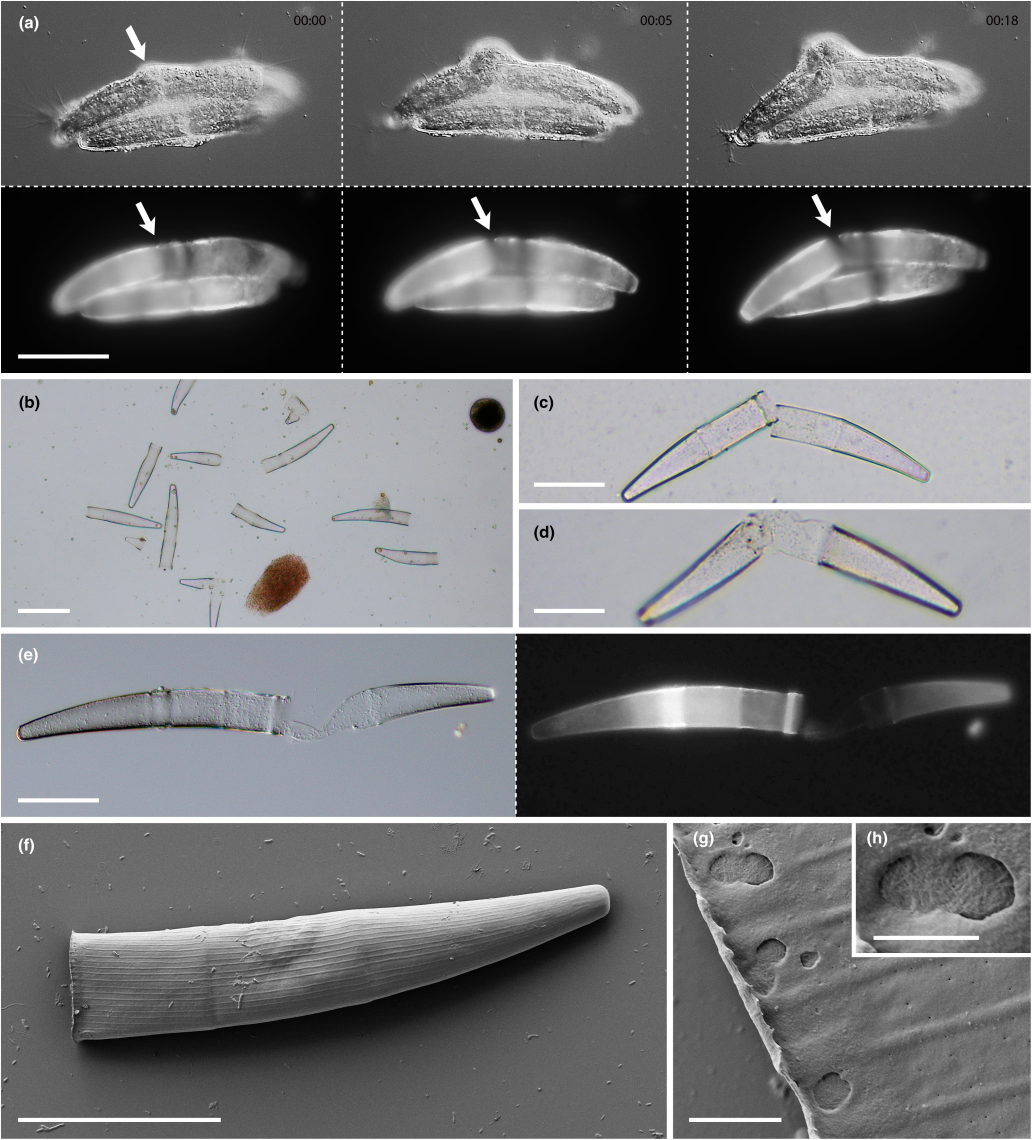
Cell wall cleavage by *Strigomyxa ruptor* and structure of algal remnants; DIC and fluorescence (a, e), brightfield (b–d), and SEM (f–h). (a) Time series of Calcofluor White‐stained *Closterium* cell in *S*. *ruptor*. Arrows indicate emerging cell wall opening. (b) Late‐stage culture of *S*. *ruptor* with bisected remnant cell walls of *Closterium intermedium* (strain C250_SH). (c) *C. intermedium* (strain C035a) remnant with both cell halves in proximity. (d) *C. intermedium* (strain C450) remnant with amorphous cell wall remains between cell wall halves. (e) Calcofluor White‐stained *C. striolatum* (strain C459) remnant showing no visible fluorescence between the cell halves. (f, g) SEM micrographs of bisected *C. intermedium* (strain C250_SH) at different magnifications revealing discrete erosion zones at the rim. Scale bars: 50 μm (a, c–f), 100 μm (b), 2 μm (g), 1 μm (h).

To learn more about the cleavage of engulfed *Closterium* cells, we fed *S. ruptor* with Calcofluor White‐stained algae and documented the cell wall fluorescence during the feeding act. This revealed a very narrow and well‐defined cell wall opening emerging on one side of the engulfed alga (Figure 4a). This opening and the initial burst of algal protoplasm was not directly associated with the bending of the algal halves, as this occurred later. Instead, the algal outline stayed more or less the same. The fluorescence data further indicated that the cell wall cleavage occurred at zones of reduced Calcofluor White signal (Figure 4a), which presumably were characterized by a reduced cell wall thickness (and potentially strength). This zonation corresponds to the age of the cell wall material and is related to the growth pattern of *Closterium* cells (Hogetsu & Shibaoka, 1978). After exocytosis, most of the remnant cell walls appeared bisected with straight edges (Figure 4b). Even though these remnants were frequently found in close association with one another they appeared clearly separated (Figure 4c). However, about 10–15% of remnant cell walls remained visibly connected by some amorphous material (Figure 4d), in particular those of larger *Closterium* strains (e.g., *C. striolatum* strain C459 and *C. intermedium* strain C450). These zones did not show any Calcofluor White signal, suggesting that they were low in or devoid of cellulose (Figure 4e). Scanning electron micrographs of empty *Closterium intermedium* cell walls (strain C250_SH) revealed that the rim close to the cleavage zone was relatively unstable as it frequently collapsed during preparation. Furthermore, we observed localized cell wall erosions in this area (Figure 4f–h), which were absent on the other parts of the cell wall.

### 3.4 Formation and characteristics of digestive cysts

**FIGURE 5:**
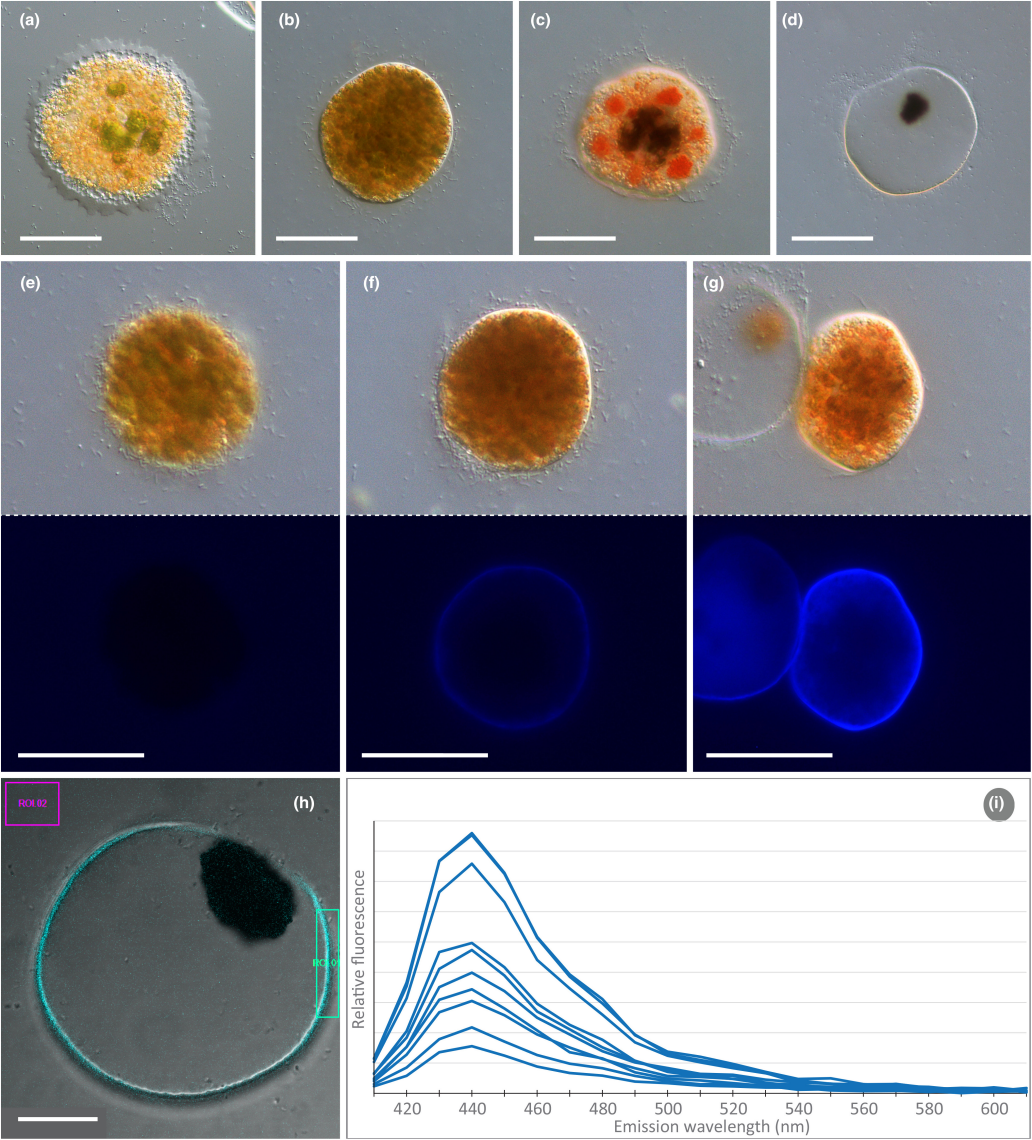
Development and characteristics of digestive cysts; DIC (a–d, h), DIC and fluorescence (e–g). (a) Forming digestive cyst with green food inclusions and hyaline fringe. (b) Early to mid‐stage digestive cyst green‐brown food inclusions. (c) Late‐stage digestive cyst with orange‐red aggregations around a central vacuole with algal remains. Note the pronounced velum surrounding the cyst. (d) Empty cyst with refractive wall and dark brown food remnants in form of a single pellet. (e–g) Blue autofluorescence of young (e), intermediate (f), and late‐stage (g) cysts. (h) Empty digestive cyst with marked scanning areas for λ‐scans. (i) Autofluorescence emission spectra of 10 digestive cysts of strain SR.01 captured with λ‐scans (excitation at 405 nm). Scale bars: 50 μm (a–g), 15 μm (h).

After the feeding phase, the amoebae became stationary and formed a cell wall. Early stages were indicated by a hyaline fringe (Figure 5a), which produced the outermost cell wall (velum). The cells then transitioned into compact digestive cysts of about 50–150 μm with several food inclusions dispersed in the cytoplasm (Figure 5b). Over time, these inclusions turned brown as the cyst matured, and at later stages were collected into a large central vacuole (Figure 5c). During these stages, the cysts turned bright orange and developed distinct aggregations of red cytoplasmic globules, which surrounded the central vacuole with undigested algal remains. The digestive cysts exhibited a pronounced, irregular velum, loosely surrounding an inner smooth cyst wall (Figure 5c). The digestion of the algal food took 48–72 h. Subsequently, a single trophozoite emerged from the cyst, leaving behind an empty, refractive cyst wall of about 1 μm in thickness and dark brown, globular food remnants (Figure 5d). The cell division did not occur in the cyst but in free, motile trophozoites through movement in two separate directions (Video 1). Furthermore, we observed a remarkable blue autofluorescence of the digestive cyst walls, which appeared to increase with the age of the cysts (Figure 5e–g). This fluorescence could be induced by ultraviolet radiation (370–400 nm) and short‐wavelength blue light (405 nm). Spectral measurements of the emitted fluorescence (λ‐scans) with a confocal laser scanning microscope revealed that the emitted light from the cysts had its peak wavelength at about 440 nm (Figure 5h,i).

### 3.5 Phylogenetic position

**FIGURE 6:**
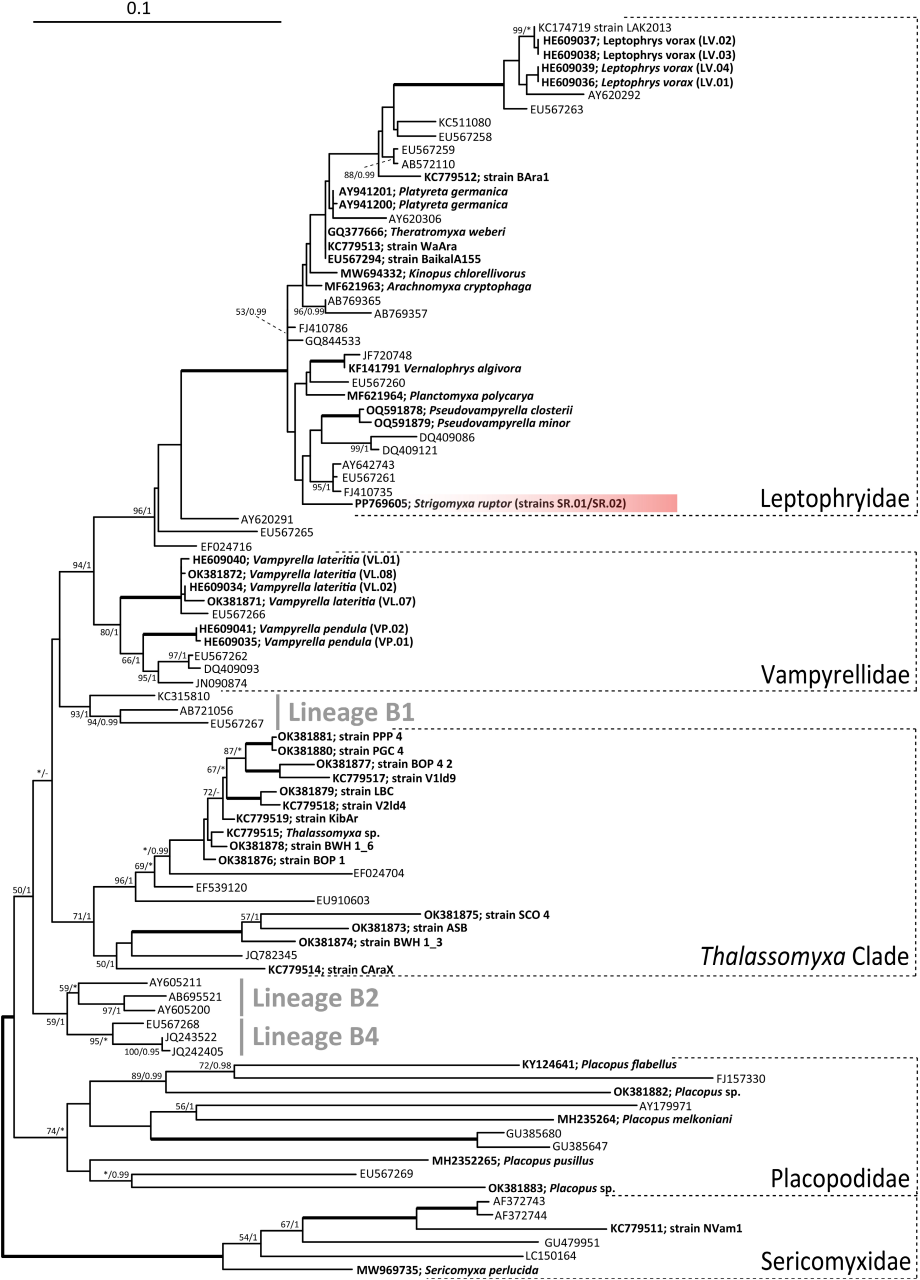
Maximum likelihood phylogeny of the Vampyrellida inferred from SSU rRNA gene sequences (92 sequences, 1498 sites, GTR + Γ + I model). The best tree of 100 inferences was rooted with the deepest branching clade (Sericomyxidae) and shows bootstrap support values and posterior probabilities at the branches (ML/BI), except for branches with full support (bold) or support <50/0.9 (*). Hyphens (−) denote branches that were not present in the tree resulting from BI. Sequence names with associated phenotypic information (cultures or imaged cells) are in bold. The scale bar corresponds to 0.1 expected substitutions per site.

We obtained near full‐length SSU rRNA gene sequences of the two studied strains of *S. ruptor*. These sequences had a sequence identity of 100% and, as revealed by BLAST searches, showed a close affinity to vampyrellid amoebae, with >93% identity to *Theratromyxa weberi* (GQ377666), *Kinopus chlorellivorus* (MW694332), *Platyreta germanica* (AY941201), and other species. Hence, we added the sequence of *S. ruptor* to the most recently published Vampyrellida dataset (Suthaus & Hess, 2023) and inferred phylogenetic trees. The best ML tree resolved all known vampyrellid lineages, but with varying support (Figure 6). Bayesian Inference showed similar results as indicated by the posterior probabilities. *Strigomyxa ruptor* was confidently placed in the family Leptophryidae, which was well supported (bootstrap support 100%; posterior probability 1). The new isolates branched with some environmental sequences from freshwater‐fed systems (lakes and soil) and the recently described genus *Pseudovampyrella* (sequence identity with *S. ruptor*: max. 94.46%). However, as previously reported, the internal branching of the Leptophryidae remained largely unresolved with single gene data (Hess, 2017b).

## 4 DISCUSSION

The two new vampyrellid strains are indistinguishable concerning their phenotypic characteristics and exhibit the identical SSU rRNA gene sequence, suggesting that they are conspecific. According to our molecular phylogeny, they belong to the family Leptophryidae and cluster relatively close to the genus *Pseudovampyrella*, though without significant support. This is an interesting finding, as the known *Pseudovampyrella* species, *P. closterii*, and *P. minor*, are specific predators of *Closterium* species (Suthaus & Hess, 2023). Likewise, our feeding experiments with *S. ruptor* suggest that this species is a specialist predator of the same genus of common moorland desmids, overlapping in prey range with *P. closterii*. However, there are distinctive differences between *Strigomyxa* and *Pseudovampyrella*, namely, the absence of motile granules on the filopodia and the feeding process. *Pseudovampyrella* species do not phagocytize entire algal cells, but extract the cell contents after annular perforation of the algal wall, thereby representing archetypical protoplast feeders (Suthaus & Hess, 2023). Furthermore, the genetic difference in the SSU rRNA gene of *Strigomyxa* and *Pseudovampyrella* species exceeds 5% and is therefore comparable to that between other leptophryid genera (e.g., *Pseudovampyrella* and *Theratromyxa*). Given these phenotypic and genetic differences, we are confident in introducing a new genus for the studied vampyrellids (diagnosis below).

**FIGURE 7:**
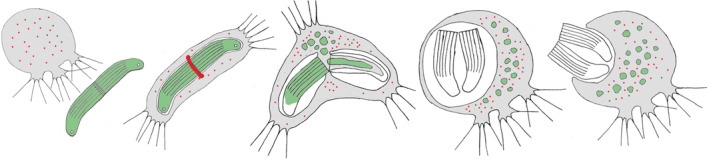
Schematic drawing of the feeding strategy of *Strigomyxa ruptor*, here named “internal protoplast extraction and cell wall regurgitation”.

The most remarkable feature of *S. ruptor* is the feeding process, which is unique among known vampyrellid species. The cell phagocytizes entire algal cells as observed in many other phylogenetically diverse vampyrellids, for example, in the genera *Leptophrys* (Cienkowski, 1865), *Vernalophrys* (Gong et al., 2015), *Kinopus* (Zhang et al., 2022), *Planctomyxa* (Hess, 2017b), *Thalassomyxa* (Grell, 1985), and *Sericomyxa* (More et al., 2021). However, the listed vampyrellids perform the free capture strategy, in which entire prey organisms undergo digestion during the digestive cyst stage (Hess & Suthaus, 2022). In *Strigomyxa*, the prey is not digested as is, but opened and subsequently extracted by internal pseudopodial action. This leads to the segregation of the nutritious algal cytoplasm and the remnant algal walls, the latter of which are then collected in a single vacuole and finally discarded by exocytosis (Figure 7). This process – reminiscent of pellet casting in owls – happens in a single eukaryotic cell and exemplifies the extraordinary complexity of prey manipulation found in some protists. It also represents a new feeding strategy of vampyrellid amoebae, here termed “internal protoplast extraction and cell wall regurgitation.”

At first sight, it appears that *Strigomyxa* breaks the algal cells in half, raising the question of how the process works. Our detailed analysis of the feeding process revealed the following sequence of events: Ingestion, formation of a red band around the algal circumference, sudden eruption of algal cytoplasm, bending of the algal cell halves, and phagocytosis of algal cell contents. Two observations indicate that the algal wall is ruptured by enzymatic instead of mechanic action: (i) Cytoplasm erupts from the algal cell, while the two cell halves are still in the original position; (ii) the initial eruption happens exactly at the position of the red band of granular cytoplasm, while there is no visible mechanical structure (which would likely act on the terminal portion of the *Closterium* cell). In addition, the scanning electron micrographs of regurgitated *Closterium* cell halves revealed cell wall erosions close to the cleavage site, which are absent in intact algal cells. From the evolutionary point of view, an enzymatic cell wall dissolution seems likely, as various vampyrellid species are able to lyse cell walls of different biochemistry in different patterns (Hess, 2017b; Suthaus & Hess, 2023). Already several decades ago, it has been hypothesized that lytic enzymes play a role in the feeding act of vampyrellids (Lloyd, 1926; Old et al., 1985), and much more recently, it was shown that another rhizarian protoplast feeder, *Orciraptor agilis*, employs a GH5_5 endoglucanase to degrade algal walls (Moye et al., 2022; Suthaus & Hess, 2023). The band of red, granular cytoplasm forming around *Strigomyxa's* prey is certainly tied to the feeding process and might contain vesicles with cell wall‐degrading enzymes that are targeted to a narrow zone at the algal wall. Interestingly, late‐stage digestive cysts exhibit conspicuous accumulations of red globules, indicating that the substances needed for the feeding act are produced in advance.

And yet, there is an additional, mechanical component to the feeding process. *Strigomyxa* produces a relatively narrow, slit‐like cell wall opening, which differs in its dimensions from the roundish perforations found in *Vampyrella*, *Pseudovampyrella*, and *Placopus* species (Hess, 2017a; Hess et al., 2012; More et al., 2019; Suthaus & Hess, 2023). To access the bulky cell contents of *Closterium* species, the algal halves need to be bent apart. This can be seen when the surrounding vacuole expands after the eruption of algal cytoplasm: The vacuole transforms into an internal compartment for prey manipulation. Opposite the cleavage site of the alga, the food vacuole remains attached to the *Closterium* cell wall, thereby forming a “cytoplasmic brace,” presumably fixing the alga in place. As the vacuole expands, the cleaved *Closterium* cell bends apart, so that a feeding pseudopodium can access the contents. In contrast to all known vampyrellids and other amoeboid protists, the feeding pseudopodium is formed internally and sent into a membrane‐bound compartment. This compartment is formed by phagocytosis, like regular food vacuoles in other organisms, but has exceptional functions. Certainly, the amoeba needs to assemble relevant cytoskeletal elements, presumably the acto‐myosin system, at this vacuole and generate forces that are otherwise atypical for food vacuoles. As the alga‐containing (primary) vacuole of *Strigomyxa* supports pseudopodium development and subsequent formation of regular food vacuoles (which undergo digestive processes), it seems to preserve some characteristics of the plasma membrane.

Finally, we discovered a conspicuous blue autofluorescence of the digestive cyst walls, which increases with cyst age. The chemical composition of the vampyrellid cyst wall is yet unknown, despite speculations on a cellulosic nature (Cienkowski, 1865; Lloyd, 1926). The blue fluorescence points to specialized metabolites that are deposited in the wall by *Strigomyxa*. In the plant world, several classes of organic compounds, for example, phenolics, fluoresce upon excitation by short wavebands such as ultraviolet radiation (Donaldson, 2020). In many cases, they function as sunscreens as they absorb and dissipate excess solar energy (e.g., Schmitz‐Hoerner & Weissenböck, 2003; Stelzner et al., 2019). Our microscopy observations of *S. ruptor* trophozoites revealed that this species is very photosensitive, by far exceeding the sensitivity of other vampyrellid species (unpublished observations). As a predator of moorland desmids from shallow ponds, *S. ruptor* is likely exposed to high levels of solar radiation. We hypothesize that the blue autofluorescence observed in the digestive cyst wall stems from yet uncharacterized, organic compounds that act as a sunscreen during the immotile life history stage of the amoeba.

Overall, the unexpected behavior and cellular details discovered in a new vampyrellid taxon, *Strigomyxa ruptor*, highlight the value of culture‐based biodiversity exploration of protists. Careful observation supported by computer‐assisted techniques such as time‐lapse microscopy is the key to uncover the natural history of such enigmatic species, and to encounter phenomena that inspire future in‐depth studies.

### TAXONOMY

**Vampyrellida** West, 1901.

**Leptophyridae** Hess et al., 2012.

***Strigomyxa*** gen. nov.

**LSID:** urn:lsid:zoobank.org:act:856A7FE2‐D29A‐4C1C‐A132‐32C3433F5B06.

**Etymology:** *στρίξ* (*stríx*) [Ancient Greek] = screecher, owl; *μύξα* (*múksa*) [Ancient Greek] = mucus, slime. The name *Strigomyxa* (feminine gender) refers to the owl‐like “regurgitation” of algal cell walls, reminiscent of pellet casting.

**Description:** Orange‐red, multinucleate vampyrellids with hyaline filopodia, which feed on whole algal cells by internal protoplast extraction and regurgitation of emptied cell walls.

**Type species:** *Strigomyxa ruptor* sp. nov.

***Strigomyxa ruptor*** sp. nov.

**LSID:** urn:lsid:zoobank.org:act:4DE615E2‐2CD3‐48DB‐854D‐7AE9AD7AA507.

**Etymology:** *ruptor* [Latin] = breaker. The epithet refers to the feeding strategy of the species, wherein it appears to break *Closterium* cells.

**Description:** Trophozoites with granular, orange‐red coloration. Cell outline variable in motion on substrate, frequently sheet‐like with multidirectional filopodia but also spherical with unidirectional filopodia. Typical cell bodies range from 50 to 150 μm in size. Filopodia are hyaline and tapering with basal branching, up to 70 μm long. Trophozoites are multinucleate with spherical nuclei of 2 μm in diameter. Feeds on *Closterium* spp. through uptake of whole algal cells, internal protoplast extraction, and regurgitation of emptied cell walls. Typical digestive cysts round to ovoid in outline, 50–150 μm in diameter, with a pronounced velum. Young digestive cysts greenish due to prey inclusions, later cysts orange with aggregations of red granules around a central digestive vacuole.

**Differential diagnosis:** Differs from all known Vampyrellida by its feeding strategy. Differs from *Pseudovampyrella* species by the absence of refractive granules, cell size, and the size of nuclei.

**Type material:** A permanent slide (aldehyde/osmium tetroxide fixed cells for DIC microscopy), constituting the name‐bearing hapantotype (article 73.3, ICZN), has been deposited in the “Protists Collection” at the Department of Life Sciences of the Natural History Museum in London (Cromwell Road, London, U.K.) with registration number NHMUK 2024.7.30.1. Note that the preparation contains algal cells (prey), which are not part of the type material.

**Type generating strain:** SR.01.

**Sequence of type generating strain (SSU rRNA gene):** PP769605.

**Type habitat and locality:** Organic sediment of moorland pond, nature reserve “Thielenbruch und Thurner Wald,” Cologne, Germany; 50.987959, 7.078928.

## AUTHOR CONTRIBUTIONS

**Andreas Suthaus:** Conceptualization (equal); investigation (lead); visualization (lead); writing – original draft (lead). **Sebastian Hess:** Conceptualization (equal); funding acquisition (lead); resources (lead); supervision (lead); visualization (supporting); writing – review and editing (lead).

## FUNDING INFORMATION

This work was funded by the German Research Foundation through the priority program SPP 1991 (Taxon‐Omics: New approaches for discovering and naming biodiversity), grant 447190101, and through the Emmy Noether program, grant 417585753 (both to S.H.).

## CONFLICT OF INTEREST STATEMENT

The authors declare no conflict of interest.

## Data Availability

The genetic data that support the findings of this study have been made publicly available and can be accessed via GenBank (https://www.ncbi.nlm.nih.gov/), Accession number PP769605.
